# Fractional Burgers Fluid Flow Due to Metachronal Ciliary Motion in an Inclined Tube

**DOI:** 10.3389/fphys.2019.00588

**Published:** 2019-05-16

**Authors:** Amer Bilal Mann, Sidra Shaheen, Khadija Maqbool, Sébastien Poncet

**Affiliations:** ^1^Department of Mathematical Sciences, Federal Urdu University of Arts Science & Technology, Islamabad, Pakistan; ^2^Department of Mathematics & Statistics, International Islamic University, Islamabad, Pakistan; ^3^Département de Génie Mécanique, Université de Sherbrooke, Sherbrooke, QC, Canada

**Keywords:** burgers fluid, cilia, fractional Adomian decomposition method, inclined tube, long wave length approximation

## Abstract

Cilia-induced flow of fractional Burgers fluid is studied in an inclined tube for both symplectic and antiplectic wave patterns. The solution of the problem is persued under the long wave length limitation. The fractional Adomian decomposition method is employed to evaluate the pressure gradient. Mathematical expressions for the axial velocity, frictional force, pressure gradient, and stream function are obtained and the influence of the main operating parameters is discussed in detail. It is noted that the velocity profile is more dominant in the case of antiplectic metachronal waves compared to symplectic ones, which confirms former results on the better capability of antiplectic waves to transport mucus, obtained with more complex numerical solvers.

## 1. Introduction

Dutch light microscopist Antoni was the first to discover cilia in 1675 and Sharpey was the first to discuss cilia in English language in 1835. Exhaustive studies on the ciliary structures have been carried out during the nineteenth century (Sleigh et al., 1988). Cilia and flagella oscillate in a waving fashion during motion to transport fluids and propel cells (Vélez-Cordero and Lauga, 2013). Cilia motion constitutes a pivotal role in a wide variety of physiological processes, such as alimentation, circulation, locomotion, respiration, and reproduction (Maiti and Pandey, 2017). Ciliated cells can indeed be found in many human organs e.g., photoreceptor cells in retina, in hair bundles on ear, epithelial cells in the respiratory tract, in Fallopian tubes (Ashraf et al., 2018), in kidney (Guirao and Joanny, 2007), in the ependymal cells of the brain that generate cerebrospinal flow among few examples (Lieberstein, 1975). Malfunctioning of the ciliary activity may be responsible of many respiratory diseases (Rubin, 2014), like severe asthma.

As the amalgamated motion of cilia occurs, the cilia's upper layer can be seen to have a metachronal wave generated as a result of a small phase lag between neighboring cilia. This collective motion of cilia supports many physiological processes (Lieberstein, 1975; Murakami and Takahashi, 1975; Takahashi and Shingyoji, 2002). Different types of metachronal waves are classified depending upon the dynamics and strokes of the moving cilia. Symplectic beat patterns are produced if the directions of the propagative metachronal waves and the main flow are the same and antiplectic patterns are recognized when the directions of the wave propagation and the flow are opposite (Knight-Jones, 1954; Blake, 1972).

To date, many efforts have been made mathematically to understand the involvement of ciliary motion related to different biofluids, such as bronchial mucus, semen …in humans which are represented by Newtonian and non-Newtonian fluid models. Maiti and Pandey (2017) applied a numerical approach to study the flow of a power-law fluid representing semen in an axisymmetric tube (representing the efferent ducts of the male human reproductive channel) due to cilia motion and concluded that, more than only the ciliary activity, there are several other factors like the smoothness of muscles, the constant fluid secretion or the vacuum created during ejaculation, which may be responsible for the semen flow. Siddiqui et al. (2010, 2014) obtained exact solutions of cilia induced flow problems of viscous fluid and power-law fluid models representing semen in a cylindrical tube and infinite channel under the long wavelength approximation.

Mucus plays a significant role in individual's health, therefore many researchers discussed the bronchial mucus transportation due to ciliary activity (see for examples in Barton and Raynor, 1967; Ross and Corrsin, 1974; Fulford and Blake, 1986; Maqbool et al., 2016). Norton et al. (2011) developed a transport model, where the mucus, considered successively as a Doi-Edwards, Jeffrey, and Maxwell fluid, is transported as a rigid body and the metachronal wave exhibits a symplectic behavior. Vélez-Cordero and Lauga (2013) applied the regular perturbation method to solve a problem in which the tracheobronchial mucus is considered as a Carreau fluid. This solution only portrays the Newtonian effects when second-order perturbations are considered and non-Newtonian effects are captured when the perturbation analysis is pushed up to the fourth-order. Maqbool et al. (2016) considered the geometry of Siddiqui et al. (2014) and studied the mucus flow treating mucus as a Jeffrey fluid. Smith et al. (2008) noted that the most advanced model to investigate the mucociliary clearance process is the Maxwell viscoelastic model and proposed a fluid-structure interaction model to examine the complex fluid flow problem arising due to ciliary activity.

More recently, the immersed boundary (IB) method (Hao and Zhu, 2010) has been extensively applied to study biofluid flows representative of humans/animals. Dillon et al. (2007) used the IB method to examine the two dimensional flow of three cilia in a mucus layer such that the mucus layer is treated as an elastic solid instead of a viscoelastic fluid. Dauptain et al. (2008) used the IB method to examine the motion of fluid due to one row of cilia on a ctenophore *Pleurobrachia pileus*, which is commonly known as a sea gooseberry for Reynolds numbers *Re* within the range [50−200]. They found that as the beating of cilia increases, it spreads more power to the interacting fluid and this work may be considered as a guideline for solving the fluid-structure interaction problem. Very recently, Chatelin and Poncet (2016) investigated by 3D simulations the influence of the mucus viscosity, fluid height, cilia length, and beating frequency on the mucociliary process in a two-phase environment. Chateau et al. (2018) performed 3D simulations of the transport and mixing induced by beating cilia at *Re* up to 20 in a two-phase environment composed of Newtonian fluids using a coupled IB/lattice Boltzmann method.

All reported studies (Barton and Raynor, 1967; Ross and Corrsin, 1974; Fulford and Blake, 1986; Dillon et al., 2007; Smith et al., 2008; Vélez-Cordero and Lauga, 2013; Maqbool et al., 2016) confirmed the fact that more efforts are still required in terms of scientific research to better understand the internal flow structure due to cilia motion and their interaction with the surrounding fluid. In the present work, one will focus on the mucociliary clearance process. It is well-known now that bronchial mucus exhibits complex rheological properties: stress relaxation, tensile stresses, shear thinning, yielding stress, and thixotropic behavior (see in Lafforgue et al., 2017, 2018). Though the advanced numerical solvers developed by Chatelin and Poncet (2016) and Chateau et al. (2018) considered the two-phase character of the problem and the behavior of each single cilium, such approaches do not account for the rheology of mucus. Moreover, simulating the mucociliary clearance process in human airways remains very challenging for such methods due to the multiscale character of the problem: from the micrometer scale when considering each individual cilium to the decimeter scale when looking at the main air flow within the trachea. So analytical approaches or simplified models like the envelope model are still deemed necessary if one wants to simulate the complete problem. The present paper is an attempt to demonstrate that analytical solutions obtained by the Adomian decomposition method can provide useful informations regarding the mucociliary clearance process. Momani and Odibat (2006) used successfully the Adomian decomposition method to solve a time-fractional Navier–Stokes equation in a tube and demonstrated both the efficiency and simplicity of its method. In this paper, bronchial mucus is considered as a fractional Burgers fluid. Motion is generated by linear pressure produced by the tips of the moving cilia under the long wavelength and low Reynolds number approximations (Shapiro et al., 1969). Various illustrations highlighting the effects of the most important parameters are also sketched. Another motivation of the present paper is that the literature is scarce on fractional fluid models (see the monograph of Oldham and Spanier, 1974 for example).

## 2. Mathematical Model

**Figure 1:**
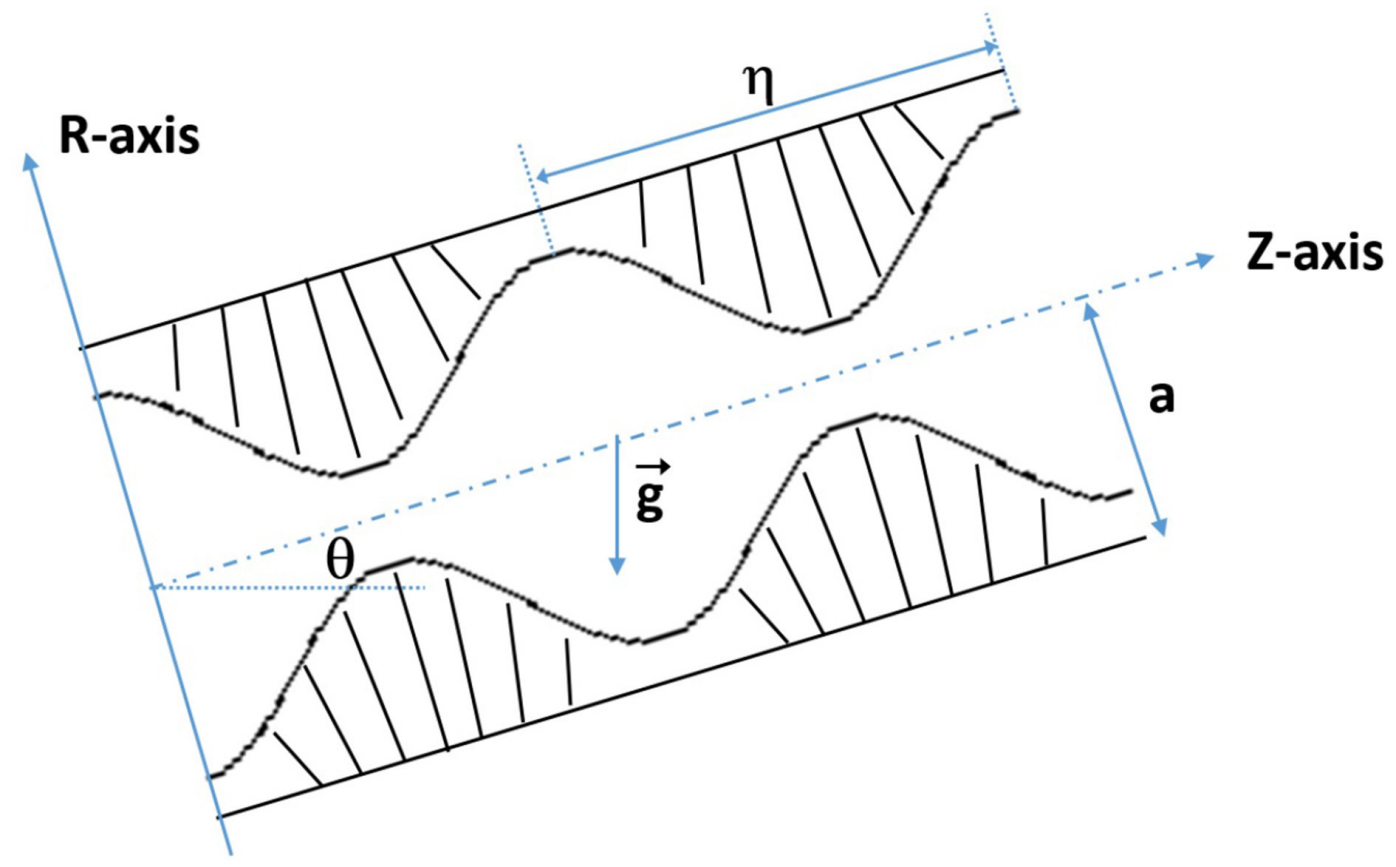
Schematic view of the inclined tube with cilia.

The fluid motion characteristics of an incompressible fractional Burgers fluid in a ciliary tube having an inclination angle θ are considered. The metachronal wave and inclined tube move with the same speed *c* to the right as shown in Figure 1.

The cilia tips follow an elliptical path as suggested by Maqbool et al. (2016) and Siddiqui et al. (2010, 2014), which can be represented by

(1) $$\begin{array}{r} \hat{l}\left(\hat{Z},\hat{t}\right)=\hat{R}=a+a\epsilon \cos\left[\frac{2\pi }{\eta }\left(\hat{Z}-c\hat{t}\right)\right], \end{array}$$

(2) $$\begin{array}{r} \hat{k}\left(\hat{Z},Z_{0},\hat{t}\right)=\hat{Z}=Z_{0}+a\epsilon \alpha^{*}\sin\left[\frac{2\pi }{\eta }\left(\hat{Z}-c\hat{t}\right)\right], \end{array}$$

where Equations (1, 2) are the parametric equations representing the cilia motion in which *a* is the mean tube radius, *c* is the wave speed, $\hat{t}$ is time, *Z*_0_ is the reference position of cilia, α^*^ is the eccentricity of ellipse, ϵ is a dimensionless varying parameter and η is the wavelength of the metachronal wave. One assumes that the fluid and adjacent cilia tips have the same velocity resulting in a no-slip condition. Therefore, the axial and radial velocities can be written as

(3) $$\begin{array}{r} \hat{W}=\frac{\partial \hat{Z}}{\partial \hat{t}}{|}_{{}_{Z=Z_{0}}}=\frac{\partial \hat{k}}{\partial \hat{t}}+\frac{\partial \hat{k}}{\partial \hat{Z}}\frac{\partial \hat{Z}}{\partial \hat{t}}=\frac{\partial \hat{k}}{\partial \hat{t}}+\frac{\partial \hat{k}}{\partial \hat{Z}}\hat{W}, \end{array}$$

and

(4) $$\begin{array}{r} \hat{U}=\frac{\partial \hat{R}}{\partial \hat{t}}{|}_{{}_{Z=Z_{0}}}=\frac{\partial \hat{l}}{\partial \hat{t}}+\frac{\partial \hat{l}}{\partial \hat{Z}}\frac{\partial \hat{Z}}{\partial \hat{t}}=\frac{\partial \hat{l}}{\partial \hat{t}}+\frac{\partial \hat{l}}{\partial \hat{Z}}\hat{W}. \end{array}$$

Introducing Equations (1, 2) into Equations (3, 4) yields

(5) $$\begin{array}{r} \hat{W}=\frac{-\left(\frac{2\pi }{\eta }\right)\left[ac\alpha^{*}\epsilon \cos\left\{\left(\frac{2\pi }{\eta }\right)\left(\hat{Z}-c\hat{t}\right)\right\}\right]}{1-\left(\frac{2\pi }{\eta }\right)\left[ac\alpha^{*}\epsilon \cos\left\{\left(\frac{2\pi }{\eta }\right)\left(\hat{Z}-c\hat{t}\right)\right\}\right]}, \end{array}$$

and

(6) $$\begin{array}{r} \hat{U}=\frac{\left(\frac{2\pi }{\eta }\right)\left[ac\alpha^{*}\epsilon \sin\left\{\left(\frac{2\pi }{\eta }\right)\left(\hat{Z}-c\hat{t}\right)\right\}\right]}{1-\left(\frac{2\pi }{\eta }\right)\left[ac\alpha^{*}\epsilon \cos\left\{\left(\frac{2\pi }{\eta }\right)\left(\hat{Z}-c\hat{t}\right)\right\}\right]}, \end{array}$$

where $\hat{U}$ and $\hat{W}$ are the radial and axial velocity components. The wave and fixed frames are related through the transformations

(7) $$\begin{array}{r} \hat{R}=\hat{r},\;\hat{U}=\hat{u},\;\hat{W}=\hat{w}-c,\;\hat{Z}=\hat{z}-c\hat{t}, \end{array}$$

and

(8) $$\begin{array}{r} \hat{P}\left(\hat{Z},\hat{R},\hat{T}\right)=\hat{p}\left(\hat{z},\hat{r}\right), \end{array}$$

where $\hat{P}\left(\hat{p}\right),$ $\hat{R}\left(\hat{r}\right)$, and $\hat{Z}\left(\hat{z}\right)$ are the pressure, radius, and axial position in the fixed (wave) frame. The extra stress tensor for fractional Burgers' fluid is given as

(9) $$\begin{array}{r} \left(1+\lambda_{1}^{\alpha }\frac{\partial^{\alpha }}{\partial t^{\alpha }}+\lambda_{2}^{2\alpha }\frac{\partial^{2\alpha }}{\partial t^{2\alpha }}\right)\Gamma =\left(1+\lambda_{3}^{\beta }\frac{\partial^{\beta }}{\partial t^{\beta }}\right)\overset{\cdot }{\varkappa }, \end{array}$$

where Γ is the shear stress tensor, $\overset{\cdot }{\varkappa }$ is the strain rate, λ_*i*_(*i* = 1, 2, 3) are constitutive parameters, α and β are the fractional derivative and integral defined as Hilfer (2000)

(10) $$\begin{array}{r} D^{\alpha }\left[q\left(p\right)\right]=\frac{1}{\Gamma \left(1-\alpha \right)}\int_{0}^{p}q\left(t\right){\left(p-t\right)}^{-\alpha }dt,\;0<\alpha <1, \end{array}$$

and

(11) $$\begin{array}{r} J^{\alpha }\left[q\left(p\right)\right]=\frac{1}{\Gamma \left(\alpha \right)}\int_{0}^{p}q\left(t\right){\left(p-t\right)}^{\alpha -1}dt,\;0<\alpha <1. \end{array}$$

with 0 ≤ α ≤ 1. The fractional Burgers fluid model reduces to fractional Oldroyd B model for λ_2_ = 0 and the classical viscous model can be obtained if λ_*i*_ = 0(*i* = 1, 2, 3).

The velocity components for the fractional Burgers' fluid model for an inclined tubular flow should satisfy the following equations

(12) $$\begin{array}{r} \rho \left(\hat{u}\frac{\partial \hat{w}}{\partial \hat{r}}+\hat{w}\frac{\partial \hat{w}}{\partial \hat{z}}\right)=-\frac{\partial \hat{p}}{\partial \hat{z}}+\frac{1}{\hat{r}}\frac{\partial }{\partial \hat{r}}\left(\hat{r}\tau_{rz}\right)+\frac{\partial \tau_{zz}}{\partial \hat{z}}-\rho g\sin\theta , \end{array}$$

(13) $$\begin{array}{r} \rho \left(\hat{u}\frac{\partial \hat{u}}{\partial \hat{r}}+\hat{w}\frac{\partial \hat{u}}{\partial \hat{z}}\right)=-\frac{\partial \hat{p}}{\partial \hat{r}}+\frac{1}{\hat{r}}\frac{\partial }{\partial \hat{r}}\left(\hat{r}\tau_{rr}\right)-\frac{\tau_{\theta \theta }}{\hat{r}}+\frac{\partial \tau_{zr}}{\partial \hat{z}}-\rho g\cos\theta . \end{array}$$

(14) $$\begin{array}{r} \frac{\partial \hat{u}}{\partial \hat{r}}+\frac{\hat{u}}{\hat{r}}+\frac{\partial \hat{w}}{\partial \hat{z}}=0, \end{array}$$

where *g* is the gravitational acceleration, $\hat{u}$ and $\hat{w}$ are the radial and axial velocity components, τ_*ij*_ are the shear stress tensor components and ρ is the fluid density. To solve the problem, the following non-dimensional parameters are introduced

(15) $$\begin{array}{c} z^{*}=\frac{\hat{z}}{\lambda },\;r^{*}=\frac{\hat{r}}{a},\;u^{*}=\frac{\hat{u}}{\beta c},\;w^{*}=\frac{\hat{w}}{c},\;h^{*}=\frac{\hat{h}}{a},\;p^{*}=\frac{a\beta }{c\mu }\hat{p},\\ \beta^{*}=\frac{a}{\eta },\;\tau_{rz}^{*}=\frac{a}{\mu c}\tau_{rz,}\;Re=\frac{\rho ca}{\mu },\;Fr=\frac{c^{2}}{ga},\\ \lambda_{1}=\frac{c\lambda_{1}}{\eta },\;\lambda_{2}=\frac{c^{2}\lambda_{2}}{\eta^{2}},\;\lambda_{3}=\frac{c\lambda_{3}}{\eta },\;t^{*}=\frac{c\hat{t}}{\eta }, \end{array}$$

where *η*, *a*, *c*, and *μ* denote the wavelength, tube radius, wave speed, and dynamic viscosity, respectively, *Re* and *Fr* are the Reynolds and Froude numbers while β^*^ is the wave number. Under the long wavelength and low Reynolds number approximations, the flow may be considered as a Stokes flow. Thus, during the non-dimensionalizing of Equations (12, 13) with the help of Equation (15), we have ignored the terms involving β^*^, β^*2^, β^*3^, and *Re*, *Re*^2^, *Re*^3^ …but terms involving *Re*/*Fr* are retained as the orders of *Re* and *Fr* numbers are the same. Equations (8–14) (after dropping hats) simplify to the following non-dimensional forms

(16) $$\begin{array}{r} \frac{\partial p}{\partial r}=0, \end{array}$$

(17) $$\begin{array}{l} \left(1+\lambda_{1}^{\alpha }\frac{\partial^{\alpha }}{\partial t^{\alpha }}+\lambda_{2}^{2\alpha }\frac{\partial^{2\alpha }}{\partial t^{2\alpha }}\right)\left(\frac{\partial p}{\partial z}+\frac{Re}{Fr}\text{ sin}\theta \right)=\\ \left(1+\lambda_{3}^{\beta }\frac{\partial^{\beta }}{\partial t^{\beta }}\right)\left(\frac{\partial^{2}w}{\partial r^{2}}+\frac{1}{r}\frac{\partial w}{\partial r}\right), \end{array}$$

with appropriate boundary conditions given as

(18) $$\begin{array}{l} u=u\left(h\right)=2\pi \varepsilon \sin\left(2\pi z\right)+\beta^{*}{\left(2\pi \varepsilon \right)}^{2}\alpha^{*}\sin\left(2\pi z\right)\cos\left(2\pi z\right)\\ \text{ at }r=\pm h, \end{array}$$

(19) $$\begin{array}{r} w=w\left(h\right)=-1-2\pi \varepsilon \alpha^{*}\beta^{*}\cos\left(2\pi z\right),\text{at }r=\pm h, \end{array}$$

where

(20) $$\begin{array}{r} h\left(z\right)=1+\varepsilon \cos\left(2\pi z\right) \end{array}$$

(21) $$\begin{array}{r} \frac{\partial w}{\partial r}\left(r=0\right)=0, \end{array}$$

The following two equations are used to determine the initial guesses required by the Adomian decomposition method

(22) $$\begin{array}{rcl} \frac{\partial p}{\partial z} & = & 0, \end{array}$$

(23) $$\begin{array}{rcl} \frac{d}{dt}\left(\frac{\partial p}{\partial z}\right) & = & 0\text{ at }t=0. \end{array}$$

Integrating Equation (17) with respect to *r* and using the boundary condition (Equation 21), one gets

(24) $$\begin{array}{l} \;\left(1+\lambda_{1}^{\alpha }\frac{\partial^{\alpha }}{\partial t^{\alpha }}+\lambda_{2}^{2\alpha }\frac{\partial^{2\alpha }}{\partial t^{2\alpha }}\right)\left(\frac{\partial p}{\partial z}+\frac{Re}{Fr}\sin\theta \right)\frac{r^{2}}{2}\\ =r\left(1+\lambda_{3}^{\beta }\frac{\partial^{\beta }}{\partial t^{\beta }}\right)\frac{\partial w}{\partial r}. \end{array}$$

Further integrating Equation (24) and applying the boundary conditions (Equations 18, 19) will yield

(25) $$\begin{array}{l} \;\left(1+\lambda_{3}^{\beta }\frac{\partial^{\beta }}{\partial t^{\beta }}\right)w\left(r\right)=\frac{1}{4}\left(r^{2}-h^{2}\right)\left(1+\lambda_{1}^{\alpha }\frac{\partial^{\alpha }}{\partial t^{\alpha }}+\lambda_{2}^{2\alpha }\frac{\partial^{2\alpha }}{\partial t^{2\alpha }}\right)\\ \times \left(\frac{\partial p}{\partial z}+\frac{Re}{Fr}\sin\theta \right)+\left(1+\lambda_{3}^{\beta }\frac{\partial^{\beta }}{\partial t^{\beta }}\right)w\left(l\right). \end{array}$$

The results for a Newtonian fluid in a horizontal tube can be deduced using the limits λ_*i*_ → 0 for *i* = 1, 2, 3 and θ → 0, therefore one gets

(26) $$\begin{array}{r} w\left(r\right)=\frac{1}{4}\left(r^{2}-h^{2}\right)\frac{\partial p}{\partial z}+w\left(h\right) \end{array}$$

The volume flow rate is defined as

(27) $$\begin{array}{r} q=2\int_{0}^{h}rwdr, \end{array}$$

which, in light of Equation (24), becomes

(28) $$\begin{array}{l} \left(1+\lambda_{3}^{\beta }\frac{\partial^{\beta }}{\partial t^{\beta }}\right)q=+h^{2}\left(1+\lambda_{3}^{\beta }\frac{\partial^{\beta }}{\partial t^{\beta }}\right)w\left(l\right)\\ +\frac{-h^{4}}{8}\left(1+\lambda_{1}^{\alpha }\frac{\partial^{\alpha }}{\partial t^{\alpha }}+\lambda_{2}^{2\alpha }\frac{\partial^{2\alpha }}{\partial t^{2\alpha }}\right)\left(\frac{\partial p}{\partial z}+\frac{Re}{Fr}\sin\theta \right), \end{array}$$

so that *q* the dimensional volume rate and *Q* the dimensionless volume flow rate in the fixed frame are related as

(29) $$\begin{array}{r} \mathbb{Q}=2\int_{0}^{l}wrdr=2\int_{0}^{l}\left(w-1\right)rdr=q-h^{2}. \end{array}$$

The mean volume flow rate $\bar{\mathbb{Q}}$ can be calculated using the time period *T* in Equation (30)

(30) $$\begin{array}{r} \bar{\mathbb{Q}}=\frac{1}{T}\int_{0}^{T}\mathbb{Q}dt^{*}=q-1-0.5\epsilon^{2}. \end{array}$$

Equation (26) in view of Equation (29) gives

(31) $$\begin{array}{l} \frac{\partial^{2\alpha }}{\partial t^{2\alpha }}\left(\frac{\partial p}{\partial z}\right)+\frac{\lambda_{1}^{\alpha }}{\lambda_{2}^{2\alpha }}\frac{\partial^{\alpha }}{\partial t^{\alpha }}\left(\frac{\partial p}{\partial z}\right)+\frac{1}{\lambda_{2}^{2\alpha }}\left(\frac{\partial p}{\partial z}\right)=\\ -\frac{Re}{Fr}\sin\theta \left(\frac{1}{\lambda_{2}^{2\alpha }}+\frac{\lambda_{1}^{\alpha }}{\lambda_{2}^{2\alpha }}\frac{t^{-\alpha }}{\Gamma \left(1-\alpha \right)}+\frac{t^{2\alpha }}{\Gamma \left(1-2\alpha \right)}\right)\\ \;\frac{-8}{\lambda_{2}^{2\alpha }}\left(1+\lambda_{3}^{\beta }\frac{\partial^{\beta }}{\partial t^{\beta }}\right)\frac{\bar{Q}+1+0.5\epsilon^{2}-l^{2}w\left(h\right)}{h^{4}} \end{array}$$

The stream function *ψ* in the wave frame is computed with the help of Equations (25, 28, 31) as

(32) $$\begin{array}{r} \psi =\frac{r^{4}-2r^{2}h^{2}}{2h^{4}}\left(h^{2}w\left(h\right)-\bar{Q}-1-0.5\epsilon^{2}\right)-\frac{r^{2}}{2}w\left(h\right). \end{array}$$

## 3. Solution Methodology

Equation (31) can be simplified as

(33) $$\begin{array}{l} \;\frac{\partial^{2\alpha }l}{\partial t^{2\alpha }}+\frac{\lambda_{1}^{\alpha }}{\lambda_{2}^{\alpha }}\frac{\partial^{\alpha }l}{\partial t^{\alpha }}+\frac{1}{\lambda_{2}^{2\alpha }}l=\\ -\frac{Re}{Fr}\sin\theta \left(\frac{1}{\lambda_{2}^{2\alpha }}+\frac{\lambda_{1}^{\alpha }}{\lambda_{2}^{2\alpha }}\frac{t^{-\alpha }}{\Gamma \left(1-\alpha \right)}+\frac{t^{2\alpha }}{\Gamma \left(1-2\alpha \right)}\right)\\ \;\left(1+\lambda_{3}^{\beta }\frac{\partial^{\beta }}{\partial t^{\beta }}\right)A, \end{array}$$

where $l\left(z,t\right)=\frac{\partial p}{\partial z}$ with the initial conditions

(34) $$\begin{array}{rcl} l\left(z,0\right) & = & 0,\\ \frac{\partial l\left(z,0\right)}{\partial t} & = & 0, \end{array}$$

and

(35) $$\begin{array}{r} A=\frac{-8}{\lambda_{2}^{2\alpha }}\frac{\bar{Q}+1+0.5\epsilon^{2}-h^{2}w\left(h\right)}{h^{4}}, \end{array}$$

so that Equation (32) will take the form

(36) $$\begin{array}{r} l\left(z,t\right)=-l^{2\alpha }\left(\frac{\lambda_{1}^{\alpha }}{\lambda_{2}^{2\alpha }}\frac{\partial^{\alpha }f}{\partial t^{\alpha }}+\frac{1}{\lambda_{2}^{2\alpha }}f-\phi \left(t\right)\right), \end{array}$$

where

(37) $$\begin{array}{l} \phi \left(t\right)=A\left[1+\lambda_{3}^{\beta }\frac{t^{-\beta }}{\Gamma \left(1-\beta \right)}\right]-\frac{Re}{Fr}\sin\theta \\ \;\left(\frac{1}{\lambda_{2}^{2\alpha }}+\frac{\lambda_{1}^{\alpha }}{\lambda_{2}^{2\alpha }}\frac{t^{-\alpha }}{\Gamma \left(1-\alpha \right)}+\frac{t^{2\alpha }}{\Gamma \left(1-2\alpha \right)}\right) \end{array}$$

An infinite series solution for *l*(*z, t*) by using the Adomian decomposition method (Adomian, 1994; Babolian and Biazar, 2002) is given by

(38) $$\begin{array}{r} l\left(z,t\right)=\overset{\infty }{\underset{n=0}{\sum }}l_{n}\left(z,t\right). \end{array}$$

where *l*_0_, *l*_1_, *l*_2_, *l*_3_, …*l*_*n*+1_ are determined as

(39) $$\begin{array}{r} l_{0}=0, \end{array}$$

(40) $$\begin{array}{l} l_{1}=A\frac{t^{2\alpha }}{\Gamma \left(1+2\alpha \right)}+\lambda_{3}^{\beta }\frac{t^{2\alpha -\beta }}{\Gamma \left(1+2\alpha -\beta \right)}+\frac{Re}{Fr}\sin\theta \\ \;\left(\frac{1}{\lambda_{2}^{\alpha }}\frac{t^{2\alpha }}{\Gamma \left(2\alpha +1\right)}+\frac{\lambda_{1}^{\alpha }}{\lambda_{2}^{\alpha }}\frac{t^{\alpha }}{\Gamma \left(\alpha +1\right)}+1\right), \end{array}$$

(41) $$\begin{array}{l} l_{2}=A\left[\frac{t^{2\alpha }}{\Gamma \left(1+2\alpha \right)}+\lambda_{3}^{\beta }\frac{t^{2\alpha -\beta }}{\Gamma \left(1+2\alpha -\beta \right)}\right]\\ \;-A\frac{\lambda_{1}^{\alpha }}{\lambda_{2}^{2\alpha }}\left[\frac{t^{3\alpha }}{\Gamma \left(1+3\alpha \right)}+\lambda_{3}^{\beta }\frac{t^{3\alpha -\beta }}{\Gamma \left(1+3\alpha -\beta \right)}\right]\\ \;-A\frac{1}{\lambda_{2}^{2\alpha }}\left[\frac{t^{4\alpha }}{\Gamma \left(1+4\alpha \right)}+\lambda_{3}^{\beta }\frac{t^{4\alpha -\beta }}{\Gamma \left(1+4\alpha -\beta \right)}\right]\\ \;+\frac{\lambda_{1}}{\lambda_{2}^{2\alpha }}\frac{Re}{Fr}\sin\theta \left[\frac{1}{\lambda_{2}^{2\alpha }}\frac{t^{3\alpha }}{\Gamma \left(1+3\alpha \right)}+\frac{\lambda_{1}^{\alpha }}{\lambda_{2}^{2\alpha }}\frac{t^{2\alpha }}{\Gamma \left(1+2\alpha \right)}+\frac{t^{\alpha }}{\Gamma \left(\alpha +1\right)}\right]\\ \;+\frac{1}{\lambda_{2}^{2\alpha }}\frac{Re}{Fr}\sin\theta \frac{1}{\lambda_{2}^{2\alpha }}\frac{t^{4\alpha }}{\Gamma \left(1+4\alpha \right)}+\frac{\lambda_{1}^{\alpha }}{\lambda_{2}^{2\alpha }}\frac{t^{3\alpha }}{\Gamma \left(1+3\alpha \right)}+\frac{t^{2\alpha }}{\Gamma \left(2\alpha +1\right)}+\\ \;-\frac{Re}{Fr}\sin\theta \left[\frac{1}{\lambda_{2}^{2\alpha }}\frac{t^{2\alpha }}{\Gamma \left(1+2\alpha \right)}+\frac{\lambda_{1}^{\alpha }}{\lambda_{2}^{2\alpha }}\frac{t^{\alpha }}{\Gamma \left(\alpha +1\right)}+1\right], \end{array}$$

(42) $$\begin{array}{l} l_{3}=A\left[\frac{t^{2\alpha }}{\Gamma (1+2\alpha )}+\lambda_{3}^{\beta }\frac{t^{2\alpha -\beta }}{\Gamma (1+2\alpha -\beta )}\right]\\ \;-A\frac{\lambda_{1}^{\alpha }}{\lambda_{2}^{2\alpha }}\left[\frac{t^{3\alpha }}{\Gamma (1+3\alpha )}+\lambda_{3}^{\beta }\frac{t^{3\alpha -\beta }}{\Gamma (1+3\alpha -\beta )}\right]\\ \;+A(\frac{\lambda_{1}^{2\alpha }}{\lambda_{2}^{3\alpha }}-\frac{1}{\lambda_{2}^{2\alpha }})\left[\frac{t^{4\alpha }}{\Gamma (1+4\alpha )}+\lambda_{3}^{\beta }\frac{t^{4\alpha -\beta }}{\Gamma (1+4\alpha -\beta )}\right]\\ \;+2A\frac{\lambda_{1}^{\alpha }}{\lambda_{2}^{3\alpha }}\left[\frac{t^{5\alpha }}{\Gamma (1+5\alpha )}+\lambda_{3}^{\beta }\frac{t^{5\alpha -\beta }}{\Gamma (1+5\alpha -\beta )}\right]\\ \;+A\left[\frac{1}{\lambda_{2}^{3\alpha }}\frac{t^{6\alpha }}{\Gamma (1+6\alpha )}+\lambda_{3}^{\beta }\frac{t^{6\alpha -\beta }}{\Gamma (1+6\alpha -\beta )}\right]\\ \;-\frac{Re}{Fr}\sin\theta \left[\frac{1}{\lambda_{2}^{2\alpha }}\frac{t^{2\alpha }}{\Gamma (1+2\alpha )}+\frac{\lambda_{1}^{\alpha }}{\lambda_{2}^{\alpha }}\frac{t^{\alpha }}{\Gamma (1+\alpha )}+1\right]\\ \;+\frac{\lambda_{1}^{\alpha }}{\lambda_{2}^{2\alpha }}\frac{Re}{Fr}\sin\theta [\frac{1}{\lambda_{2}^{2\alpha }}\frac{t^{3\alpha }}{\Gamma (1+3\alpha )}+\frac{\lambda_{1}^{\alpha }}{\lambda_{2}^{2\alpha }}\frac{t^{2\alpha }}{\Gamma (1+2\alpha )}\\ \;+\frac{t^{\alpha }}{\Gamma (1+\alpha )}]\\ \;-(\frac{\lambda_{1}^{\alpha }}{\lambda_{2}^{3\alpha }}-\frac{1}{\lambda_{2}^{2\alpha }})\frac{Re}{Fr}\sin\theta [\frac{1}{\lambda_{2}^{2\alpha }}\frac{t^{4\alpha }}{\Gamma (1+4\alpha )}+\frac{\lambda_{1}^{\alpha }}{\lambda_{2}^{2\alpha }}\frac{t^{3\alpha }}{\Gamma (1+3\alpha )}\\ \;+\frac{t^{2\alpha }}{\Gamma (1+2\alpha )}]\\ \;-2\frac{Re}{Fr}\sin\theta \frac{\lambda_{1}^{\alpha }}{\lambda_{2}^{2\alpha }}[\frac{1}{\lambda_{2}^{2\alpha }}\frac{t^{5\alpha }}{\Gamma (1+5\alpha )}+\frac{\lambda_{1}^{\alpha }}{\lambda_{2}^{2\alpha }}\frac{t^{4\alpha }}{\Gamma (1+4\alpha )}\\ \;+\frac{t^{3\alpha }}{\Gamma (1+3\alpha )}]. \end{array}$$

From *l*_*n*_(*z, t*) (*n* ≽ 0), the other components can also be obtained. Finally an approximate solution of Equation (37) by truncating the series can be written as

(43) $$\begin{array}{r} l\left(z,t\right)=\underset{N\to \infty }{\lim}\phi_{n}\left(z,t\right), \end{array}$$

where

(44) $$\begin{array}{r} \phi_{n}\left(z,t\right)=\overset{N-1}{\underset{n=0}{\sum }}l_{n}\left(z,t\right). \end{array}$$

The pressure difference Δ*p* and friction force *F* across one wavelength are given by

(45) $$\begin{array}{rcl} \Delta p & = & \int_{0}^{1}\frac{\partial p}{\partial z}dz, \end{array}$$

(46) $$\begin{array}{rcl} F & = & \int_{0}^{1}\left(-h\frac{\partial p}{\partial z}\right)dz \end{array}$$

## 4. Results and Discussion

In order to evaluate the pressure rise Δ*p*, pressure gradient *dp*/*dz*, frictional force *F*, and streamlines ψ, the software Mathematica 8.0 has been used. The flow characteristics of Burgers fluids in an inclined ciliated tube are presented by controlling the fractional parameters α and β, the cilia length ϵ, and the angle of inclination θ. The Reynolds number *Re* and the Froude number *Fr* have been both fixed to 0.1. The Reynolds number based on the cilia tip speed is usually around 10^−5^ in human airways but Chateau et al. (2018) recently demonstrated that there is no significant influence of *Re* as long as it remains lower than 1.

Table 1 depicts that the flow of the fractional Burgers fluid model gives the larger magnitude of the frictional force: typically *F* is 16.5 and 38.9 times higher for the fractional Burgers fluid model compared to the Burgers and the fractional Oldroyd-B models, respectively. The pressure difference Δ*p* for the Oldroyd-B model is greater than for the fractional Burgers model, meaning that this last fluid is more ease to be transported by the mucociliary clearance process.

**Table 1:** Pressure rise, pressure gradient, and frictional force for the four rheological models considered here.

**Fluid models**	**Rheological properties**	**Δ*p***	***dp/dz***	***F***
Fractional burgers	λ_1_ = λ_2_ = λ_3_ = 1, α = β = 0.5	3.62293	−1.9542	3.46156
Burgers	λ_1_ = λ_2_ = λ_3_ = 1, α = β = 1	7.90151	−0.624569	0.209561
Fractional Oldroyd-B	λ_1_ = λ_3_ = 1, λ_2_ = 0, α = β = 0.5	7.75994	0.251982	0.0889968
Oldroyd-B	λ_1_ = λ_3_ = 1, λ_2_ = 0, α = β = 1	−7.27648	0.794008	−0.814099

*Results obtained for r = 0.1 and z = 0.5 at t = 0.1*.

**Figure 2:**
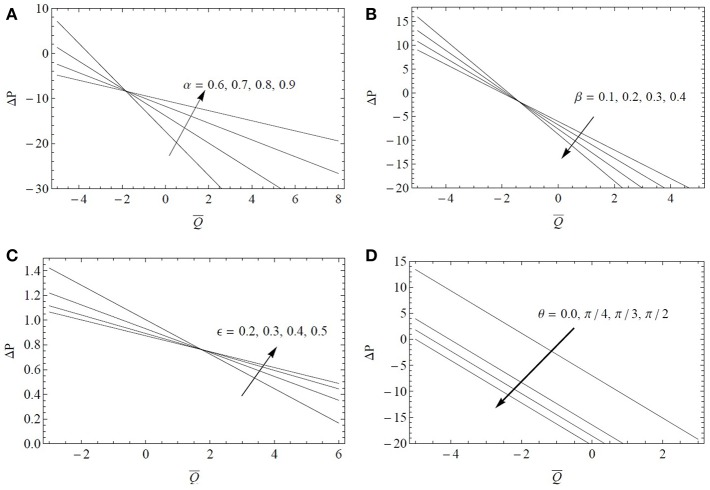
Pressure drop Δ*p* as a function of the flow rate $\bar{Q}$ for α^*^ = β^*^ = 0.4, *Fr* = 0.1 and *Re* = 0.1: **(A)** influence of the fractional parameter α for ϵ = 0.3, β = 0.6, λ_1_ = 5, λ_2_ = λ_3_ = 1, $\theta =\frac{\pi }{3}$; **(B)** influence of the fractional parameter β for ϵ = 0.3, α = 0.6, λ_1_ = 5, λ_2_ = λ_3_ = 1, $\theta =\frac{\pi }{3}$; **(C)** influence of the cilia length ϵ for α = β = 0.6, λ_1_ = 5, λ_2_ = λ_3_ = 1, $\theta =\frac{\pi }{3}$; **(D)** influence of the inclination angle θ for ϵ = 0.3, α = β = 0.6, λ_1_ = 5, λ_2_ = λ_3_ = 1.

The variations of Δ*p* with $\bar{Q}$ are examined in Figure 2 for different values of α, β, ϵ, and θ. It is found that the pressure rise Δ*p* decreases with an increase of the fractional parameter α, cilia length ϵ in the pumping expanse region (Δ*p* > 0) of the tube and an opposite trend is noted in the copumping expanse region (Δ*p* < 0) of the tube. Also in the pumping (resp. copumping) expanse region, the pressure rise increases (resp. decreases) for increasing values of the fractional parameter β. As expected increasing the cilia length or decreasing the tube radius provide similar results in terms of pressure variations but the effect is more pronounced by changing the tube radius. The ratio β/ϵ may be the relevant geometric parameter governing the transport of fluid by the mucociliary clearance process. The influence of the inclination angle θ on Δ*p* is more straightforward as shown in Figure 2D. Δ*p* increases by increasing θ in the pumping expanse region and an opposite trend is reported in the copumping expanse region. Larger pressure differences are observed for a vertical tube.

**Figure 3:**
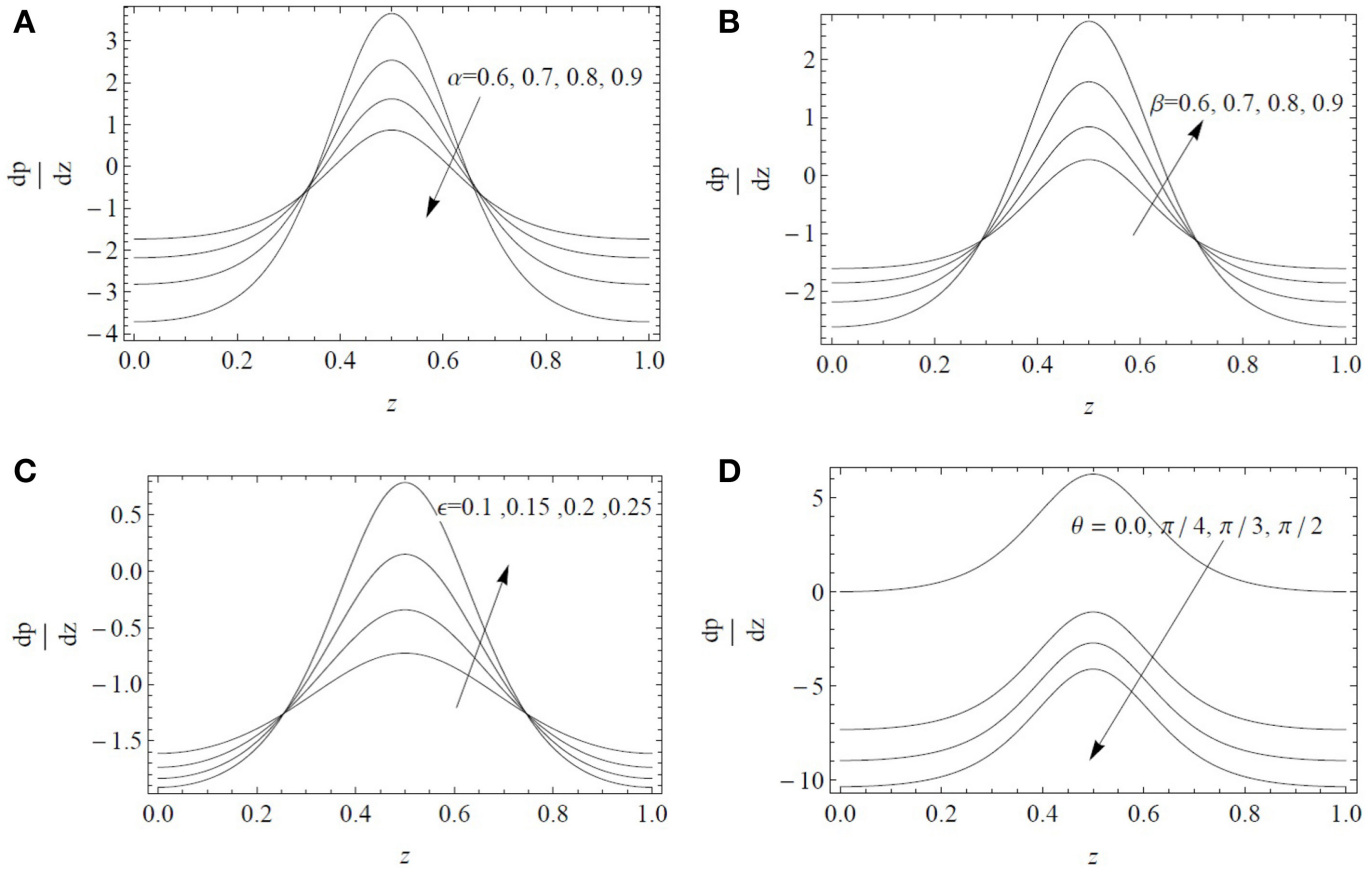
Axial pressure gradient *dp*/*dz* as a function of the axial position *z* for α^*^ = β^*^ = 0.4, *Fr* = 0.1 and *Re* = 0.1: **(A)** influence of the fractional parameter α for ϵ = 0.3, β = 0.6, λ_1_ = 5, λ_2_ = λ_3_ = 1, $\theta =\frac{\pi }{3}$; **(B)** influence of the fractional parameter β for ϵ = 0.3, α = 0.6, λ_1_ = 5, λ_2_ = λ_3_ = 1, $\theta =\frac{\pi }{3}$; **(C)** influence of the cilia length ϵ for α = β = 0.6, λ_1_ = 5, λ_2_ = λ_3_ = 1, $\theta =\frac{\pi }{3}$; **(D)** influence of the inclination angle θ for ϵ = 0.3, α = β = 0.6, λ_1_ = 5, λ_2_ = λ_3_ = 1.

The variations of the pressure gradient $\frac{dp}{dz}$ are examined in Figure 3 for different values of α, β, ϵ, and θ. It is noted that $\frac{dp}{dz}$ remains small and the fluid can flow smoothly without the application of a large pressure gradient in the expanse regions 0 ≤ *z* ≤ 0.2 and 0.8 ≤ *z* ≤ 1. On the other hand, for 0.2 < *z* < 0.8, a large amount of pressure gradient is required to maintain the flow. The magnitude of the pressure gradient increases by increasing the parameters β, ϵ, and θ. These parameters provide the resistive force to the flow thus a larger value of $\frac{dp}{dz}$ is required to maintain the fluid flow, whereas the parameter α provides the deriving force to the flow thus a smaller value of $\frac{dp}{dz}$ is required to maintain the fluid flow.

**Figure 4:**
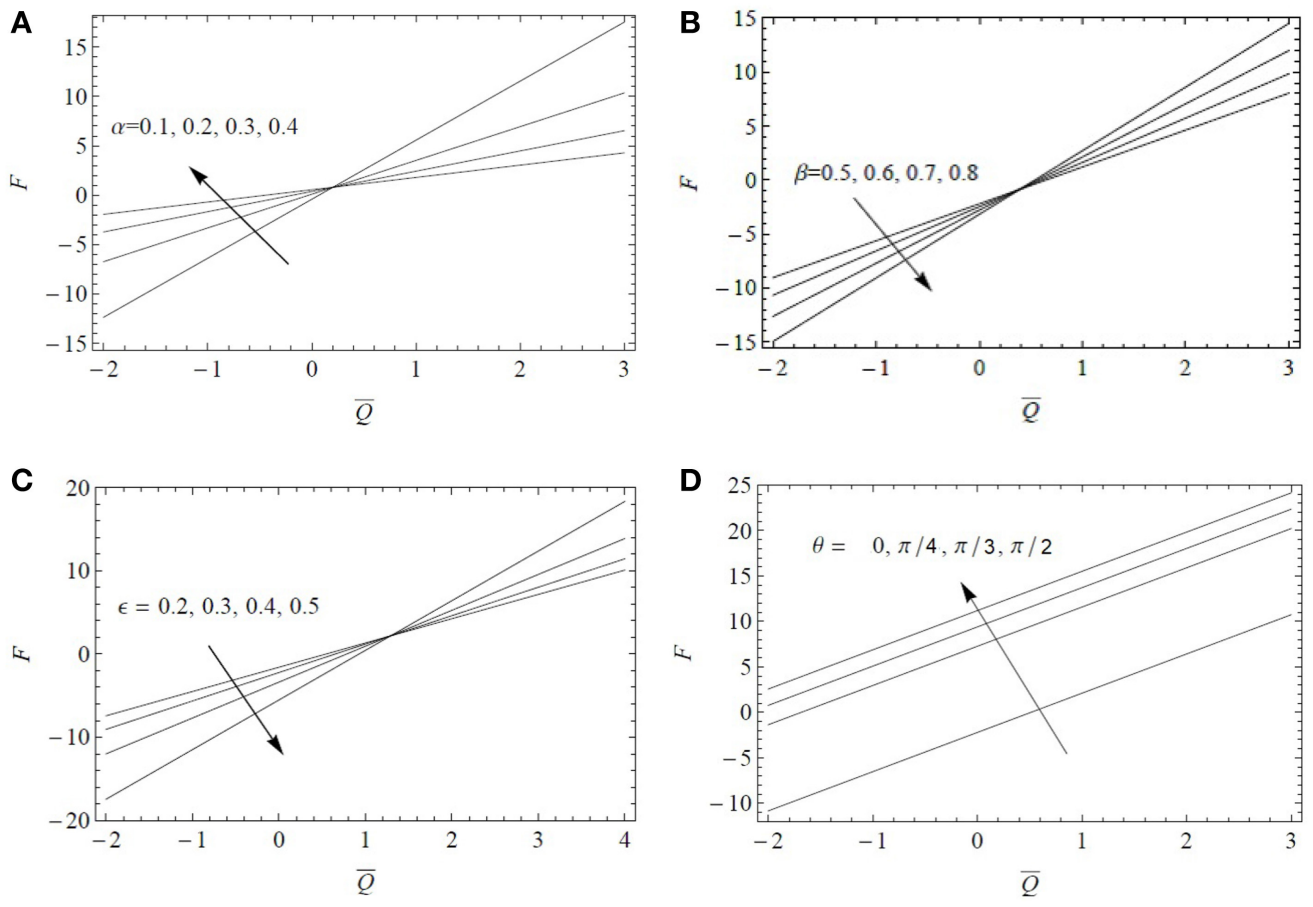
Variations of *F* as a function of the flow rate $\bar{Q}$ for *Fr* = 0.1 and *Re* = 0.1: **(A)** influence of the fractional parameter α for α^*^ = β^*^ = 0.4, ϵ = 0.3, β = 0.6, λ_1_ = 5, λ_2_ = 1, λ_3_ = 1, $\theta =\frac{\pi }{3}$; **(B)** influence of the fractional parameter β for α^*^ = β^*^ = 0.4, ϵ = 0.3, α = 0.6, λ_1_ = 5, λ_2_ = 1, λ_3_ = 1, $\theta =\frac{\pi }{3}$; **(C)** influence of the cilia length ϵ = 0.2 for α = β = 0.6, α^*^ = β^*^ = 0.6, λ_1_ = 5, λ_2_ = 1, λ_3_ = 1, $\theta =\frac{\pi }{3}$; **(D)** influence of the inclination angle θ for ϵ = 0.3, α = β = 0.6, α^*^ = β^*^ = 0.6, λ_1_ = 5, λ_2_ = 1, λ_3_ = 1.

It is observed through Figure 4 that the frictional force *F* varies linearly by increasing α, β, and ϵ and it tends to increase in magnitude by increasing β and ϵ. The magnitude of the resistive force decreases by increasing α, in the expanse region $\bar{Q}>0.5$. On the contrary, its magnitude increases in the same region when increasing β and/or ϵ. The magnitude of the frictional force increases in the expanse $\bar{Q}<1$ by increasing the tube inclination θ but an opposite trend is noted for $\bar{Q}>1$. The inclination of the tube provides then a large magnitude of *F* to oppose the flow.

**Figure 5:**
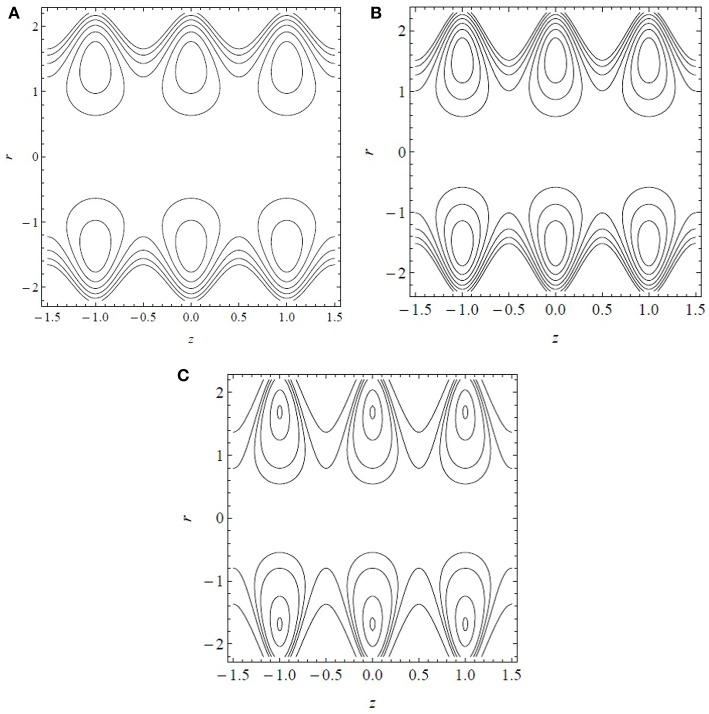
Streamline patterns for **(A)** ϵ = 0.2, **(B)** ϵ = 0.3, and **(C)** ϵ = 0.4. Results obtained for α^*^ = β^*^ = 0.7 and $\bar{Q}=1.5$.

Figure 5 displays the streamline patterns and trapping for α^*^ = β^*^ = 0.7 and $\bar{Q}=1.5$ by increasing the dimensionless cilia length ϵ. The center line symmetry bifurcates the boluses of the fluid particles circulating along the closed stream lines. The boluses are confined and move with the velocity of the metachronal waves. In Figures 5A–C, keeping all the other parameters constant, the number and size of the boluses increase by increasing ϵ. Thus, the cilia length significantly affects the flow dynamics by generating boluses in the inclined tube.

**Figure 6:**
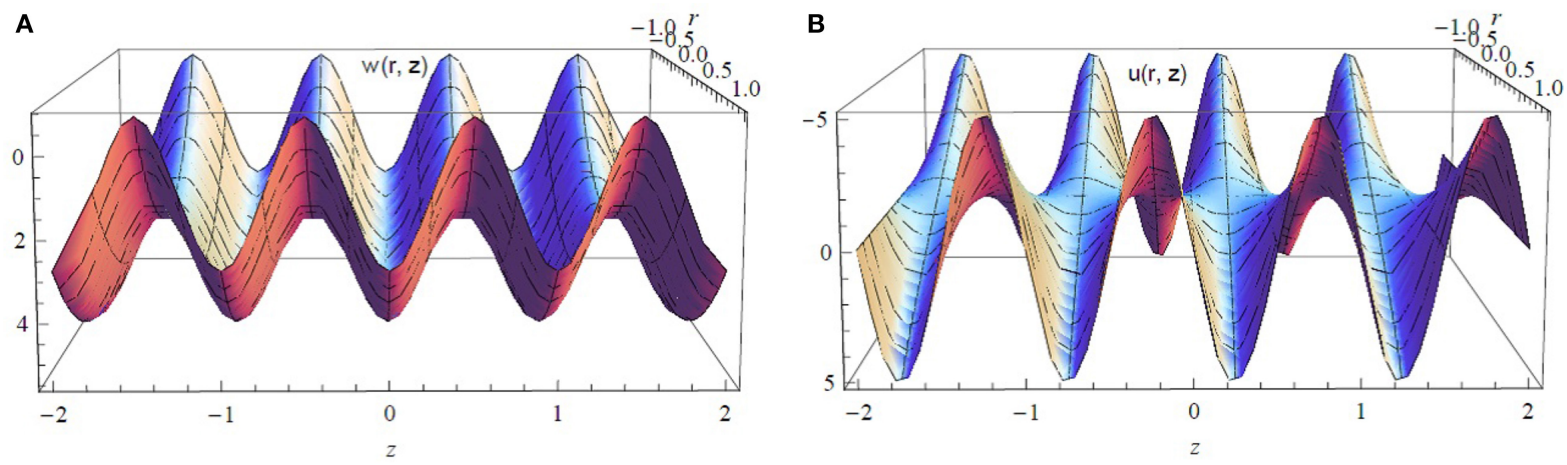
**(A)** Axial and **(B)** radial components of the velocity vector for ϵ = 0.2, α^*^ = β^*^ = 0.7, and $\bar{Q}=1.5$.

Figure 6 displays a 3D view of the axial and radial velocity components for ϵ = 0.2, α = 0.7, β = 0.7, and $\bar{Q}=1.5$. It is observed that the waves are elliptic waves and the fluid velocity is in both forward and backward directions with a symmetric behavior due to metachronism of the ciliary motion.

**Figure 7:**
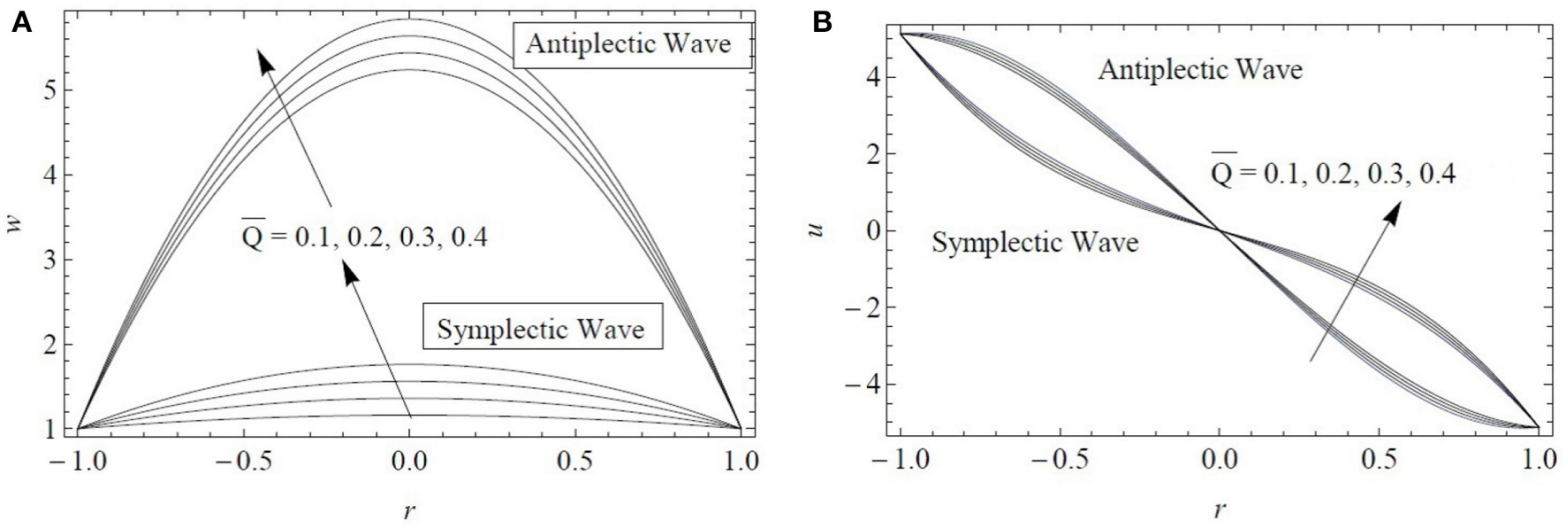
Comparison in terms of the **(A)** axial and **(B)** radial velocity components plotted at *z* = 0.75 for both antiplectic and symplectic waves. Results obtained for ϵ = 0.2, α^*^ = β^*^ = 0.7, and $\bar{Q}=1.5$.

Figure 7 displays the radial distributions of the axial and radial velocity components for four flow rates $\bar{Q}$ and both antiplectic and symplectic waves formed by the cilia tips. As expected, both components increase by increasing the flow rate. More interestingly, at any value of $\bar{Q}$, the axial velocity produced by antiplectic waves is larger than the one due to symplectic waves. The increase in the velocity profiles is also faster for antiplectic waves compared to symplectic waves. Regarding the radial velocity component, the two types of metachronal waves provide symmetric profiles in the radial direction. All in all, it confirms the former results of Chateau et al. (2018) obtained using a more advanced numerical model but for a two Newtonian fluid configuration. For a single layer of fractional Burgers fluid, antiplectic waves are also more efficient to transport fluid than symplectic waves.

## 5. Conclusions

In this paper, fractional Burgers' fluid flow in an inclined ciliated tube is examined. Using the long wavelength approximation, a semi-analytic solution is developed. Frictional force, pressure rise, pressure gradient and streamlines are plotted for different values of the main operating parameters and the main results can be summarized as follows:

- Δ*p* decreases by increasing α and ϵ in the pumping region and an opposite trend is noted in the copumping region of the tube.
- Δ*p* increases in the pumping region and decreases in the copumping region when increasing β.
- $\frac{dp}{dz}$ is insignificant for 0 ≤ *z* ≤ 0.2 and 0.8 ≤ *z* ≤ 1. On the contrary, for 0.2 < *z* < 0.8, a higher value of $\frac{dp}{dz}$ is required to maintain the flux.
- The magnitude of $\frac{dp}{dz}$ increases by increasing β, ϵ, and θ.
- It is noticed that the parameters β, ϵ, and θ greatly influence the pressure gradient whereas the parameter α provides a smaller amount of the pressure gradient to the fluid flow.
- The frictional force varies linearly when increasing α, β, and ϵ and its magnitude increases by increasing β and ϵ but it decreases in magnitude by increasing α and θ in the pumping region.
- The magnitude of the frictional force increases in the region $\bar{Q}<1$ by increasing the tube inclination θ but an opposite trend is noted in the region $\bar{Q}>1$.
- The magnitude of the pressure difference is larger for fractional generalized Burgers model in comparison to the generalized Burgers model for $\bar{Q}<0.6$ and a reverse trend is observed for $\bar{Q}>0.6$.
- A greater magnitude of the pressure gradient is needed to the fluid flow for fractional generalized Burgers fluid as compared with the generalized Burgers fluid to pump the same amount of fluid.
- The fractional generalized Burgers fluid provides a greater amount of frictional force in comparison to the generalized Burgers fluid.
- The number and size of boluses increase by increasing ϵ.
- The magnitude of the velocity profile increases significantly for antiplectic waves as compared to symplectic waves confirming that they are more efficient to transport mucus.

The fractional Adomian decomposition method appears then as a valuable tool to get useful and realistic results for the mucociliary transport process. Such method provides much faster results compared to more complex solvers (Chatelin and Poncet, 2016; Chateau et al., 2018) and could be associated to 3D flow solvers to solve the multiscale problem of the mucus clearance in the airways.

## Author Contributions

This work is the product of intellectual effort of all authors. They read and approved the final draft of the manuscript. KM and AM suggested and formulated the problem. KM, SS, and AM solved the problem jointly. SS wrote the paper. SP helped in data analysis and reviewed the manuscript.

### Conflict of Interest Statement

The authors declare that the research was conducted in the absence of any commercial or financial relationships that could be construed as a potential conflict of interest.
